# Chronische Otitis mesotympanalis beidseits

**DOI:** 10.1007/s00106-023-01302-0

**Published:** 2023-05-08

**Authors:** Andrea Sheila Büchel, Markus Jungehülsing, Tillmann Schumacher

**Affiliations:** Ernst-von-Bergmann-Klinikum, Charlottenstr. 72, 14467 Potsdam, Deutschland

In der Hals-Nasen-Ohren-Heilkunde stellen sich häufig Patient/innen mit chronischen Otitiden vor, die teilweise therapieresistent sind und unspezifische Entzündungen zeigen. Dabei sollte an eine Reihe von Systemerkrankungen und ihre Manifestation im HNO-Bereich gedacht werden.

## Fallbericht

### Anamnese

Wir berichten über eine 23-jährige Patientin, die sich erstmals im Juli 2019 mit Verdacht auf akute Otitis media mit vestibulärer Beteiligung rechts sowie chronischer Otitis mesotympanalis links vorstellte. Es erfolgte eine Parazentese beidseits sowie eine intravenöse Therapie mit Ampicillin/Sulbactam und Methylprednisolon. Bei persistierender Otorrhö und Hörminderung links erfolgte dann im August 2019 die Wiedervorstellung zur sanierenden Ohr-Op. und Mastoidektomie bei Verdacht auf ein Cholesteatom. Histologisch konnte nur eine uncharakteristische Entzündung nachgewiesen werden. Eine PCR-Diagnostik auf Mycobacterium tuberculosis aus Material aus dem linken Mastoid war negativ. Bei persistierendem Trommelfelldefekt rechts erfolgte im September 2019 die Tympanoskopie rechts. Hier konnte ebenfalls nur eine chronische, granulierende, unspezifische Entzündung nachgewiesen werden. Die beidseitige chronische Otitis media mit mesotympanalem Defekt bestand weiterhin. Zum Ausschluss einer Granulomatose mit Polyangiitis (vormals M. Wegener) wurde die Patientin im November zur rheumatologischen Diagnostik eingewiesen. Hier klagte sie dann über einen neu aufgetretenen Husten mit putridem Auswurf. In der daraufhin angefertigten Computertomographie (CT) des Thorax zeigten sich Infiltrate im linken Lungenunterlappen. Zur weiterführenden Diagnostik wurde eine bronchoalveoläre Lavage durchgeführt.

### Erhobene Befunde

**Figure 1:**
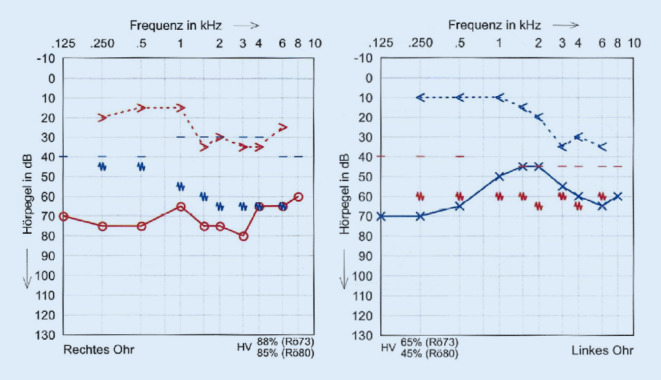

**Figure 2:**
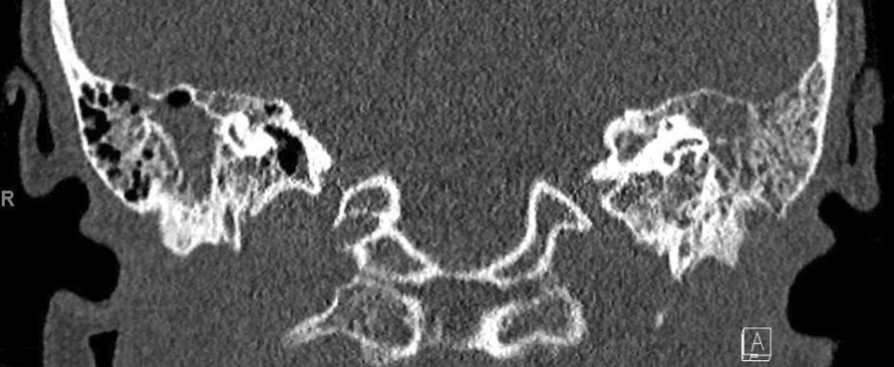

**Figure 3:**
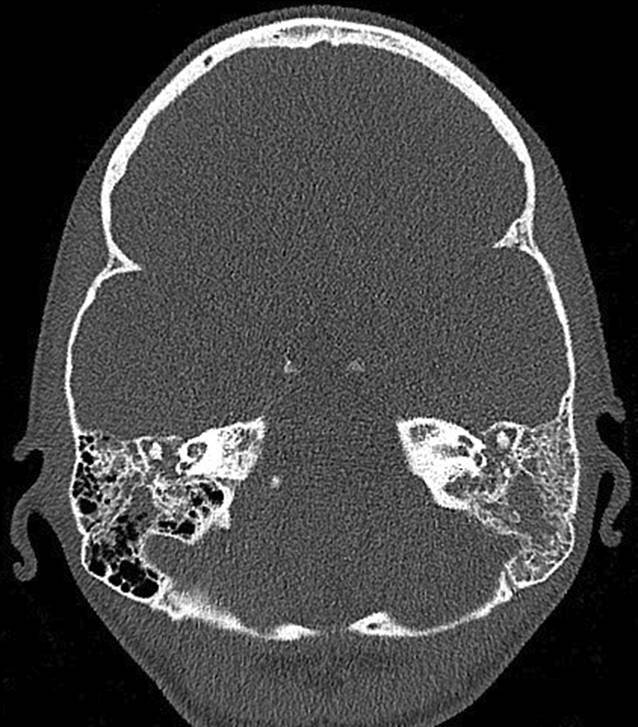

(Abb. 1, 2, 3, 4 und 5).

**Figure 4:**
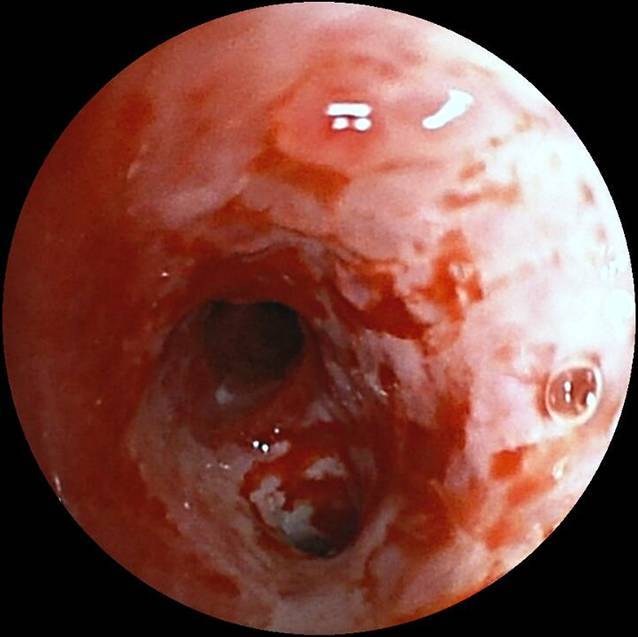

**Figure 5:**
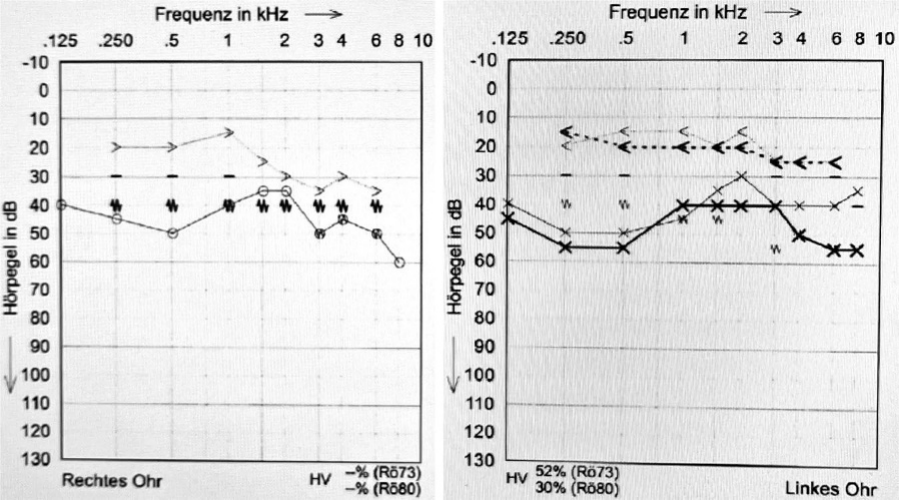

### Mikrobiologie und Laborparameter

- Ohrabstrich August 2019: steril
- Histologie August 2019: uncharakteristische Entzündung; explizit kein Hinweis auf spezifische Entzündung; kein Keimnachweis in der PCR
- Labor: kein Nachweis von c‑ANCA
- Histologie Ohr rechts September 2019: chronische, granulomatöse Entzündung,
- in der molekularpathologischen Untersuchung positiver Ausfall auf Mycobacterium-tuberculosis-Komplex-1- und -2-DNA
- Quantiferontest: negativ
- CRP und Leukozyten: normwertig
- Harnsäure: erhöht

### Kultur und PCR-Testung im Oktober 2019

In der bronchoalveolären Lavage konnten große Mengen an säurefesten Stäbchen mit positiver PCR für Mycobacterium tuberculosis nachgewiesen werden. In der Kultur zeigte sich eine Empfindlichkeit auf die Standardmedikation.

## Wie lautet Ihre Diagnose?

### Therapie und Verlauf

Die Vierfachtherapie mit Pyrazinamid, Isoniazid, Rifampicin und Ethambutol wurde im Oktober 2019 eingeleitet. Darunter zeigten sich die Symptome regredient. Die beidseitige Otorrhö sistierte, die Trommelfellperforationen rechts und links verschlossen sich spontan, und das Hörvermögen verbesserte sich leicht. Es zeigte sich eine weiterhin bestehende Schallleitungskomponente, das Innenohr zeigte nur wenig Reaktion auf die tuberkulostatische Therapie. Die Patientin konnte mit Hörgeräten beidseits ausgestattet werden. Bei progredienten Parästhesien der Oberarme erfolgte die Umstellung von Ethambutol auf Moxifloxacin einen Monat nach Therapiebeginn. Wegen der möglichen ossären Beteiligung der Mastoide wurde die Zweifachtherapie mit Isoniazid und Rifampicin für weitere 7‑10 Monate empfohlen.

### Definition

Tuberkulose ist eine chronisch granulierende Infektion mit dem Mycobacterium tuberculosis. Die Übertragung erfolgt hauptsächlich durch das respiratorische System und betrifft am häufigsten die Lungen. Sind andere Teile des Körpers betroffen, so wird diese Form als „extrapulmonale Tuberkulose“ bezeichnet [1]. Tuberkulose gehört auch heute noch zu einer der 10 tödlichsten Krankheiten in den Entwicklungsländern, kann aber auch in unseren Breitengraden vorkommen [2].

**Diagnose: **Offene Lungentuberkulose mit beidseitiger tuberkulöser Otitis media

Bei den extrapulmonalen Manifestationen zeigen sich auch Manifestationen im Hals-Nasen-Ohren-Bereich. Nach einer Metaanalyse von Qian et al. [1] zeigen sich extrapulmonale Manifestationen im Hals-Nasen-Ohren-Bereich am häufigsten an zervikalen Lymphknoten (87,9 %), gefolgt vom Larynx (8,7 %), der Befall anderer Regionen wie Pharynx, Nase und Mittelohr tritt wesentlich seltener auf (3,4 %). Eine Übertragung kann hämatogen oder auch transtubar entstehen. Die Mykobakterien können meist in der Kultur nachgewiesen werden [1, 3].

Die Therapie entspricht der der Tuberkulose mit pulmonaler Manifestation (Vierfachtherapie mit Pyrazinamid, Isoniazid, Rifampicin und Ethambutol für mindestens 2 Monate, dann Reduktion auf Zweifachkombination für 4 weitere Monate), sollte aber bei Knochenbeteiligung oder ZNS-Beteiligung auf 9–12 Monate erweitert werden [4, 5].

## Fazit für die Praxis

Tuberkulose und ihre extrapulmonalen Formen treten besonders häufig im HNO-Bereich auf, wenn auch die Tuberkulose in Deutschland derzeit eine niedrige Inzidenz aufweist. Bei untypischen und therapierefraktären Symptomen sollte auch an eine systemische Erkrankung gedacht werden. Auch eine therapieresistente exsudative Otitis media kann eine Manifestation einer Tuberkulose darstellen. Zeigt sich ein Abstrich auf das Mycobacterium tuberculosis negativ, sollte man bei therapierefraktären Symptomen auch eine molekularpathologische Untersuchung anschließen, um eine Erkrankung mit Tuberkulose endgültig auszuschließen.
